# Present State of Our Knowledge of Electricity

**Published:** 1834

**Authors:** 


16. *Present state of our knowledge of Electricity.*—In a late article on *imponderable agents* we endeavored to show that it is incumbent on all who would study the laws of animal life, to make themselves acquainted with what relates to electricity, galvanism, magnetism, light and caloric, so far at least as to appreciate the influence of these agents upon the living body both in health and disease. Under this conviction, we shall extract from the scientific journal, which contains the paper just evicerated, a short article translated from the French by professor Griscom. It presents the conclusions of professor De La Rive, on the present state of electrical science.

"1st. Two different principles are acknowledged to exist in electricity; the laws of action to which these principles give rise have been determined, both when they are isolated and at rest, and when they are in motion in order to unite. But the nature of them has not yet been determined: nothing has yet been done but to advance hypotheses which are still unsatisfactory,—such especially as that which regards them as very subtle fluids, endowed with certain distinct properties. It is probable that they are rather, both of them, different modifications of the ethereal matter which fills the universe, and whose vibrations constitute light; modifications, the nature of which cannot be known until the most intimate properties of electricity have been more thoroughly studied and ascertained.

"2d. It has been successfully determined that magnetism is only the result of natural electric currents. But what is the disposition of these currents in magnetised bodies? What is the cause which gives rise to them, and what is the reason that a very small number of bodies only is susceptible of the magnetic virtue? These are questions which cannot yet be answered.

"3d. All the sources of electricity are probably at present known; but with respect to the laws which, in each case govern its development, we are still very far from having discovered them.

"4th. The influence which bodies may exert over electricity, either when placed in its track, or interposed between it and the points towards which its exterior action is directed, has been within a short time past, studied with great care. Many curious phenomena, in relation to this influence have been discovered; some laws even have been settled; but the number of anomalies and unexplained effects is still very considerable. It is probable that in the investigation of this class of facts, means may be found of arriving at some notions with respect to the nature of electricity, and the relations which connect this agent with ponderable matter.

"5th. The effects which electricity produces on bodies are now well known; the laws to which they are subjected, are in general well determined; but their connexion with the cause which produces them rests only on hypotheses, which can boast but very little solidity, and which have lately been very much shaken. It is by an inquiry into this connexion by a study in detail of those effects, that we are to find the means of arriving at a more just idea of the nature of electricity, and of the cause of the effects to which it evidently gives rise, as well as which are perhaps erroneously ascribed to it, and which, such as chemical phenomena, have probably no other relation to it but that of being occasioned by the operation of the same agent.

"6th. Finally, after having, from its very origin, framed and demolished theories to account for the action of the Voltaic pile, philosophers are not yet united on that subject, and although at the present time, the chemical theory of this admirable apparatus may perhaps be most in vogue, it still requires the support of further observation to be generally adopted, and definitively substituted for the electromotive theory of Volta, the unsatisfactory nature of which is now fairly demonstrated.

"This brief recapitulation is sufficient to show that notwithstanding the importance of the discoveries with which electricity has been enriched, that which remains to be done in this part of physical science, is perhaps more considerable than all that has hitherto been done, since almost all its laws and all its principles are still to be discovered."

We hope that such of our western readers as are qualified and inclined to look a little into the curious relations subsisting among the various imponderable agents, and, especially made manifest by the phenomena of what is termed electro-magnetism, will peruse with care the paper of Dr. Locke, of this city, inserted in the first department of our journal. It will not only afford them instruction in this new branch of science, but show them that, with all its obscurities and intricacies, it has found a zealous and successful cultivator in the West. Such of them as may be desirous of possessing one of Dr. Locke's galvanometers, can have it made under his inspection, by applying to him.

